# Retraction: Silymarin Targets β-Catenin Signaling in Blocking Migration/Invasion of Human Melanoma Cells

**DOI:** 10.1371/journal.pone.0210344

**Published:** 2018-12-31

**Authors:** The PLOS ONE Editors

Following publication of this article [1], concerns were raised regarding Figure 5E. An area in the bottom right quadrant of the second panel (silymarin 10μg/ml) is very similar to an area in the upper left quadrant of the fourth panel (silymarin 40μg/ml). The University of Alabama at Birmingham confirmed that the original records and data needed to determine the validity of these experiments are not available.

Following a joint investigation by the Birmingham VA Medical Center and the University of Alabama at Birmingham, the institutions requested retraction of this article, as the conclusions could not be supported by available data. In line with the institutions’ investigation and recommendation, the *PLOS ONE* Editors retract this article based upon the unavailability of original data and records and the ambiguous identification of samples and treatments.

The authors did not comment on the retraction decision.
